# N_2_O formation by nitrite-induced (chemo)denitrification in coastal marine sediment

**DOI:** 10.1038/s41598-019-47172-x

**Published:** 2019-07-31

**Authors:** Julia M. Otte, Nia Blackwell, Reiner Ruser, Andreas Kappler, Sara Kleindienst, Caroline Schmidt

**Affiliations:** 1 Geomicrobiology, Center for Applied Geosciences, University of Tübingen, Tübingen, Germany; 2 Microbial Ecology, Center for Applied Geosciences, University of Tübingen, Tübingen, Germany; 3 Fertilization and Soil Matter Dynamics, Institute of Crop Science, University of Hohenheim, Stuttgart, Germany; 4 Center for Geomicrobiology, Aarhus University, Aarhus, Denmark

**Keywords:** Biogeochemistry, Biogeochemistry

## Abstract

Nitrous oxide (N_2_O) is a potent greenhouse gas that also contributes to stratospheric ozone depletion. Besides microbial denitrification, abiotic nitrite reduction by Fe(II) (chemodenitrification) has the potential to be an important source of N_2_O. Here, using microcosms, we quantified N_2_O formation in coastal marine sediments under typical summer temperatures. Comparison between gamma-radiated and microbially-active microcosm experiments revealed that at least 15–25% of total N_2_O formation was caused by chemodenitrification, whereas 75–85% of total N_2_O was potentially produced by microbial N-transformation processes. An increase in (chemo)denitrification-based N_2_O formation and associated Fe(II) oxidation caused an upregulation of N_2_O reductase (typical *nosZ*) genes and a distinct community shift to potential Fe(III)-reducers (*Arcobacter*), Fe(II)-oxidizers (*Sulfurimonas*), and nitrate/nitrite-reducing microorganisms (*Marinobacter*). Our study suggests that chemodenitrification contributes substantially to N_2_O formation from marine sediments and significantly influences the N- and Fe-cycling microbial community.

## Introduction

Nitrous oxide (N_2_O) is one of the most important long-lived greenhouse gases with an atmospheric lifetime of 131 ± 10 years^1^. N_2_O has a 265 or 298 (without or with climate-carbon feedbacks, respectively)^2^ times higher global warming potential than the same mass of CO_2_ and contributes up to 6% to the overall global radiative forcing^3^ by participating in the depletion of the stratospheric ozone layer through photochemical nitric oxide (NO) production^4^. Globally, annual N_2_O emissions are derived from soils (6.6 Tg per year), wetlands (0.17 Tg per year), rice paddies (2.8 Tg per year), wildfires and biomass burning (0.1 Tg per year), rivers (<0.6 Tg per year), lakes (<0.04 Tg per year), open oceans (3.8 Tg per year), as well as in coastal marine sediments (1.7 Tg per year)^5^. A variety of biotic and abiotic processes generate N_2_O but the specific contribution of the individual processes to the global N_2_O budget is still uncertain^6^. Processes forming N_2_O include: (1) nitrification (oxidation of ammonia to nitrate)^7^, (2) denitrification (reduction of nitrate to NO, N_2_O or N_2_)^8^ by fungi, archaea, and bacteria, (3) dissimilatory nitrate reduction to ammonium (DNRA)^9^, (4) nitrifier-denitrification (ammonia oxidation to nitrite followed by the reduction of nitrite to nitric oxide)^10,11^ and (5) nitrite-induced (chemo)denitrification, e.g. by ferrous iron (Fe(II))^12,13^. Nitrous oxide is also produced by anaerobic methane-oxidizing bacteria^14^, ammonia-oxidizing archaea^15^, and anammox bacteria^16^. It has been suggested that microbial N_2_O production is dominated by nitrification and denitrification^17^.

Microbial denitrification proceeds via several metabolic steps, which can be followed by the activity of the respective enzymes. The only known microbially mediated reduction of N_2_O is the microbial reduction to N_2_ via (a)typical *nosZ*-encoded N_2_O reductases^18,19^. Nitrate reduction can be mediated by microorganisms that couple Fe(II) oxidation to nitrate reduction (NRFeOx)^20^. Several cultures of NRFeOx have been isolated from various environments and have been shown to be involved in the emission of high levels of N_2_O^21^. Only recently it has been proven that the oxidation of Fe(II) during NRFeOx is an abiotic process stimulated by nitrite and Fe(II)^22^. This abiotic process is triggered by the biotic production of reactive nitrogen species during denitrification^22^. The rapid abiotic reduction of nitrite by Fe(II) is an important N_2_O source in nature and termed chemodenitrification^23,24^.

1 $$4{{\rm{Fe}}}^{2+}+2{{\rm{NO}}}_{2}^{-}+5{{\rm{H}}}_{2}{\rm{O}}\to 4{\rm{FeOOH}}+{{\rm{N}}}_{2}{\rm{O}}+6{{\rm{H}}}^{+}$$

Chemodenitrification could be driven by the presence of Fe(II) that is produced by heterotrophic Fe(III)-reducing microorganisms^23^, as well as by the availability of nitrite, that is produced during the reduction of nitrate by heterotrophic denitrifying bacteria^18^. The abiotic production of N_2_O via chemodenitrification has been documented in laboratory experiments via reactions involving intermediates such as hydroxylamine (NH_2_OH) and NO_2_^−^ ^25^. Hereafter, the term chemodenitrification refers to the abiotic reaction of Fe(II) and nitrite. The extent of N_2_O production via chemodenitrification versus denitrification is still poorly understood. Elevated levels of N_2_O have been observed in numerous studies examining iron- and nitrate-/nitrite-rich environments, e.g. soils^26^, hypersaline ponds and brines in Antarctica^27^, and marine coastal sediments^28–30^. However, these high levels of N_2_O have been solely attributed to microbial denitrification, potentially overlooking the important contribution of chemodenitrification to the overall N_2_O formation. Jones *et al*.^31^ showed with a purely chemical laboratory setup that the co-presence of Fe^2+^ and nitrite clearly stimulates chemodenitrification. They also provided an approach to distinguish between biotic versus abiotic contribution to N_2_O emission based on isotopic labelling. However, the actual role and potential of chemodenitrification in environmental systems remains unclear. The connection of the biogeochemical N and Fe cycle via chemodenitrification potentially impacts on the related microbial community. The production of Fe(III) minerals during chemodenitrification triggers heterotrophic Fe(III) reduction which supplies Fe^2+^, stimulating again chemodenitrification in the presence of nitrite, as well as microbial Fe(II) oxidation. Iron redox cycling is thus, strongly related to the biogeochemical N cycle. Based on microsensor measurements Wankel *et al*.^30^ could show N_2_O formation at the interface of the nitrate and Fe(III) reduction zone within marine sediments. These authors hypothesized that chemodenitrification could play a major role in N_2_O production and hinted towards connections between the biogeochemical Fe and N cycle. Still, the actual contribution of chemodenitrification to N_2_O emission across the sediment-water interface remains unknown. Therefore, the focus of our study was to quantify the chemodenitrification-based N_2_O formation potential in natural marine sediments and to investigate the potential impact on the microbial community.

We hypothesize (i) that chemodenitrification plays an important role in marine sediments and that the abiotic oxidation of Fe(II) (provided by microbial Fe(III) reduction) by nitrite (formed during heterotrophic nitrate reduction) produces significant amounts of N_2_O, and (ii) that chemodenitrification may influence the N- and Fe-cycling microbial community in marine sediments.

Here we present incubation experiments with marine organic-rich sediment from the coastal area of the Baltic Sea, Norsminde Fjord, Denmark. Based on the knowledge gaps described above, the objectives of the present study were (i) to quantify N_2_O formation during chemodenitrification in microcosm studies, and (ii) to understand the consequences of chemodenitrifying conditions on the N- and Fe-cycling microbial community.

In our study, we found that up to 15–25% of total N_2_O production (range of three independent experiments) can be caused by chemodenitrification. This elevated N_2_O formation caused an increase of N_2_O reductase (*nosZ*) transcripts and an enrichment of potential Fe(II)-oxidizers and Fe(III)-reducers, as revealed by quantitative PCR and 16S rRNA (gene) amplicon sequencing, respectively. Our study demonstrates that chemodenitrification can contribute substantially to global N_2_O formation and significantly influences the N- and Fe-cycling microbial community in marine coastal sediments.

## Results

### Nitrite-induced (chemo)denitrification in coastal marine sediment

**Figure 1:**
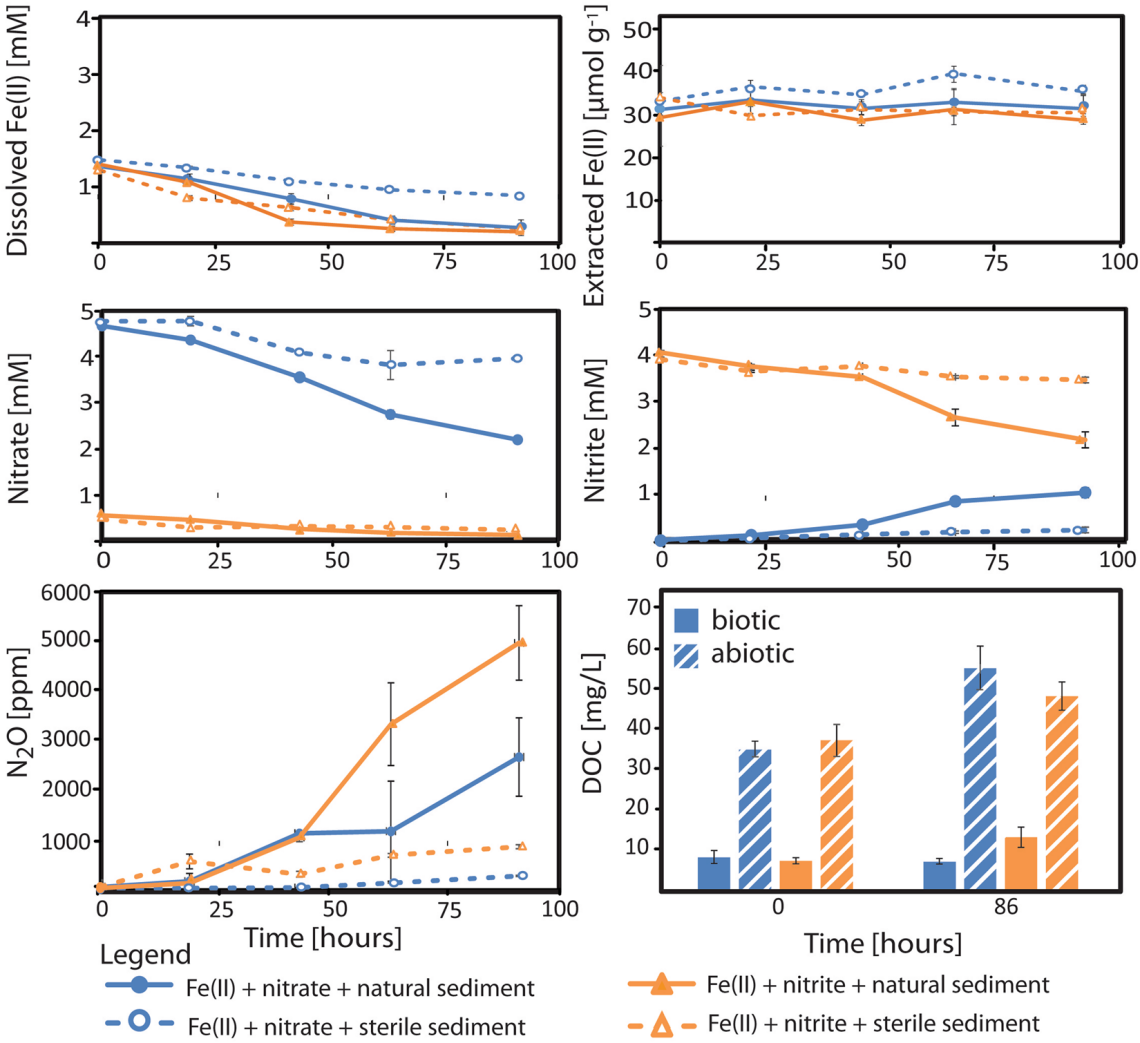
Sediment microcosms amended with dissolved Fe(II) and nitrate/nitrite. 4 mM nitrate and 2 mM FeCl_2_ (shown in blue) or 4 mM nitrite and 2 mM FeCl_2_ (shown in orange) were added to microbially active or sterilized marine Norsminde Fjord sediment collected in spring 2016 and N_2_O was quantified over time. Dissolved Fe(II) and nitrate/nitrite is shown in mM, extractable Fe(II) in μmol g^−1^ wet sediment, N_2_O in ppm and DOC in mg/L. Results shown are average of three parallel microcosm setups (standard deviation is based on biological triplicates). Norsminde Fjord water without carbonate-buffer and additives had a DOC content of 4 mg/L.

**Figure 2:**
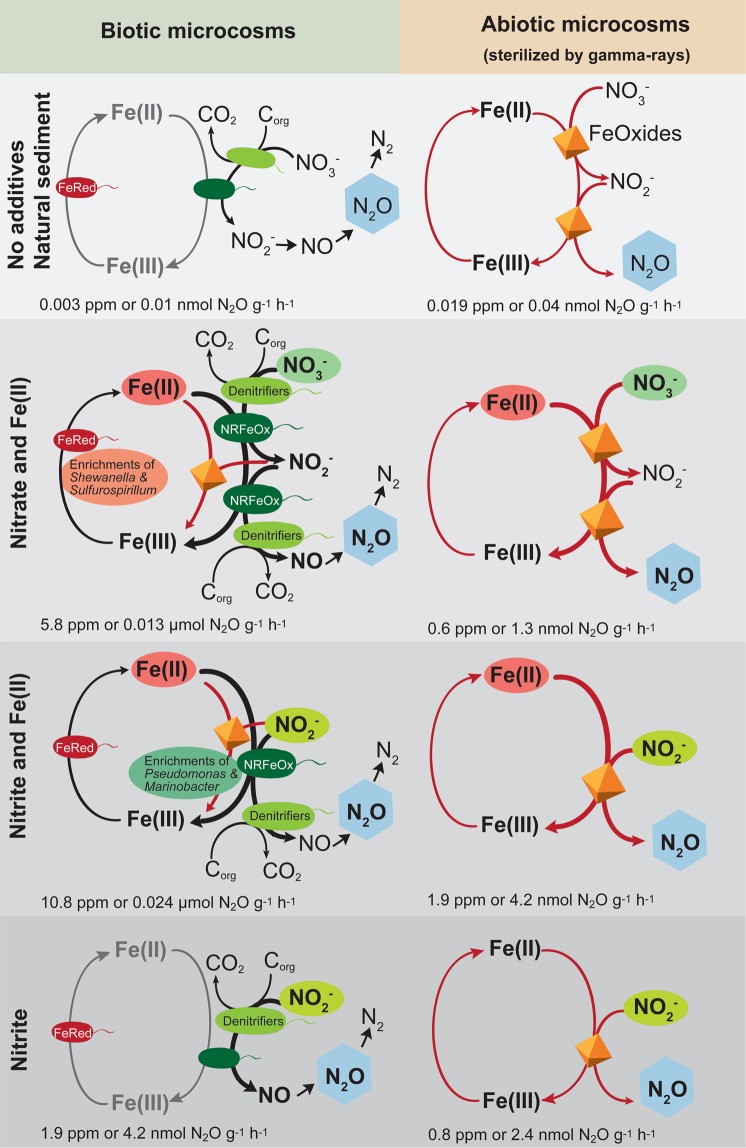
Conceptual model of the influences of nitrate or nitrite on microbial Fe-cycling and N_2_O production based on the detected N_2_O production rates [ppm g^−1^ h^−1^] or [nmol g^−1^ h^−1^]. The model is based on results of microcosm experiments with marine sediment from Norsminde Fjord amended with Fe(II) and nitrate, Fe(II) and nitrite (Fig. 1); or amended with nitrite only and native sediment (Fig. S1) (sterilized and microbially active setups). Thickness of lines and differences in symbol size indicate the relative importance. Heterotrophic denitrifiers are marked in light green, nitrate-reducing Fe(II)-oxidizers (NRFeOx) in dark green, and Fe(III)-reducers (FeRed) in red. Chemodenitrification reactions (abiotic reduction of nitrite by Fe(II), green rust or siderite) are highlighted in red. The orange mineral particle stands for iron minerals (e.g. FeOOH, green rust, siderite).

The following experiments were set up: amendment of (i) nitrate and Fe(II) (Fig. 1), (ii) nitrite and Fe(II) (Fig. 1), (iii) nitrite only (Fig. S1) to both microbially active and sterilized sediments at concentrations to quantify the maximum contribution of chemodenitrification to N_2_O formation. In addition, one setup contained only native sediment (sterile vs. microbial active) (Fig. S1). The different setups are summarized in Table S1. In microcosms containing natural sediments, nitrite and Fe(II) were continuously produced and consumed via microbial and abiotic processes, and their resulting steady-state concentrations were low. In sterilized microcosms nitrite and Fe(II) were not produced during the experiment (Fig. S1). When N substrates and Fe(II) were added to the microbially active sediment, a maximum of 2653 ± 787.0 ppm of N_2_O (nitrate addition) and 4950 ± 748.6 ppm (nitrite addition) was quantified after four days of incubation (Fig. 1). This yielded a maximum amount of 5.8 ± 1.7 ppm (with nitrate) and 10.8 ± 1.6 ppm (with nitrite) per g wet sediment per hour for the respective microcosm setup. Thus, a maximum amount of N_2_O of 0.013 ± 0.001 µmol g^−1^ h^−1^ and 0.36 ± 0.1 µg N kg^−1^ h^−1^ (nitrate addition) and 0.024 ± 0.001 µmol g^−1^ h^−1^ and 0.68 ± 0.1 µg N kg^−1^ h^−1^ (nitrite addition) was formed (for calculations see Wang *et al*.^26^) (Fig. 2). The addition of Fe(II) and nitrate to sterilized sediment only showed a low production of 0.6 ± 0.03 ppm N_2_O per g wet sediment after four days of incubation, which relates to 1.3 ± 0.1 nmol g^−1^ h^−1^. In contrast, the amendment of Fe(II) and nitrite to sterilized sediment revealed approximately three times more N_2_O production (1.9 ± 0.08 ppm N_2_O per g wet sediment per hour) which relates to 4.2 ± 0.1 nmol g^−1^ h^−1^. Thus, a maximum of 17.4 ± 6.6% of total N_2_O (result of three independent microcosm experiments) was produced abiotically via chemodenitrification.

In nitrite-amended microcosms with microbially active sediment (without Fe(II) addition, Fig. S1), 1.87 ± 0.6 ppm N_2_O was formed per g wet sediment per hour, which relates to 4.2 ± 0.2 nmol N_2_O g^−1^ h^−1^. For comparison, the sterile control setup that was amended with nitrite only produced 0.82 ± 0.4 ppm N_2_O per g wet sediment per hour, which relates to 2.4 ± 0.07 nmol g^−1^ h^−1^. Almost no N_2_O was formed in native sterile and active microcosms within four days of incubation (<0.04 nmol N_2_O g^−1^ h^−1^) (Fig. S1).

To evaluate the effect of iron and nitrogen substrates on the formation of N_2_O, we quantified the extractable and dissolved Fe(II), as well as total Fe, nitrate, nitrite, and dissolved organic matter (DOC) over time (Fig. 1). Extractable Fe(II) was relatively constant at approximately 31.7 ± 1.5 μmol wet g^−1^ in the nitrate-/nitrite- and Fe(II)-amended (both microbially active and sterile setups) (Fig. 1). Whilst dissolved Fe(II) decreased in all setups containing both microbially active sediment and an amendment of nitrate or nitrite (from 1.6 ± 0.1 mM to 0.1 ± 0.02 mM and 0.2 ± 0.03 mM, respectively), dissolved Fe(II) decreased from 1.6 ± 0.1 to 0.8 ± 0.01 mM in sterilized sediment with nitrate addition and from 1.5 ± 0.1 to 0.3 ± 0.03 mM in sterilized sediment with nitrite addition. The total extractable Fe(II) values and Fe_total_ values were both approx. 32.5 ± 0.3 μmol wet g^−1^. The nitrate concentration in microbially active sediment with nitrate addition decreased from 4.7 ± 0.02 mM to 2.2 ± 0.1 mM, probably due to nitrate reduction by denitrifying microorganisms (see Fig. S2; e.g. potential nitrate-reducers: *Sulfurimonas* and *Desulfuromonadales*). In the sterilized nitrate-amended sediments nitrate decreased to a much lower extent (from 4.7 ± 0.04 mM to 3.9 ± 0.04 mM). We observed a decrease of nitrite in nitrite-amended microbially active and sterilized setups (from 4.0 ± 0.04 mM to 2.7 ± 0.2 mM and to 3.5 ± 0.1 mM, respectively) and an increase of nitrite in nitrate-amended microbially active and in sterilized setups (from 0 to 0.9 ± 0.1 mM and 0.2 ± 0.01 mM, respectively). Furthermore, the DOC content in sterilized sediment increased significantly, probably as a result from the gamma-radiation treatment. In contrast, in microbially active nitrite and Fe(II)-amended microcosms, the DOC increased only slightly while DOC even decreased in microbially active setups that were amended with nitrate and Fe(II) (probably due to denitrification activity) (Figs 1 and 2).

### Consequences of N_2_O formation and (chemo)denitrification for gene expression specific for the nitrogen cycle

**Figure 3:**
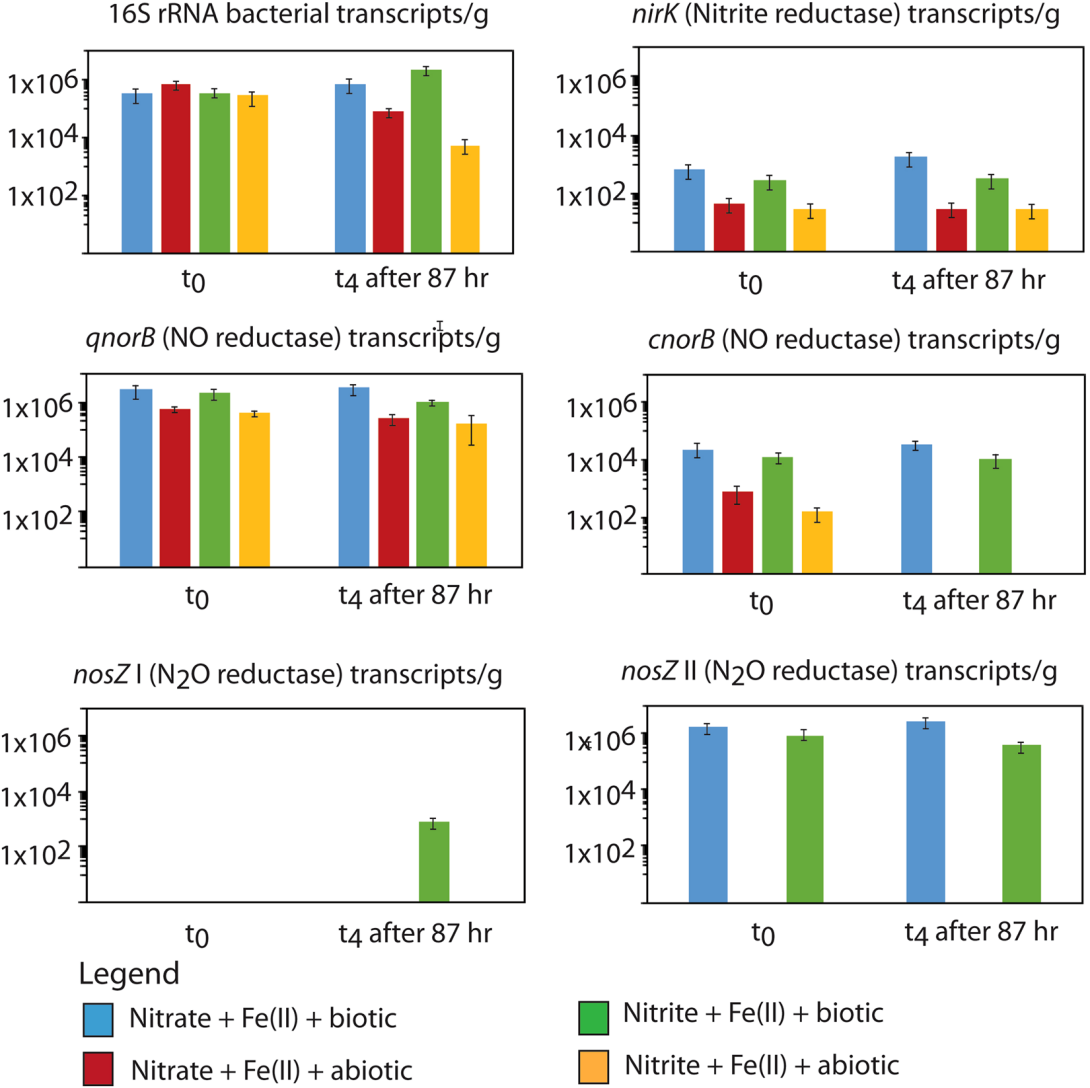
Absolute abundance of bacterial 16S rRNA and nitrogen cycle transcripts in biotic and abiotic microcosms (shown in Fig. 1). Transcripts in abiotic experiments were detectable. RNA might be stable due to inactivated RNAse by gamma-rays^41^. Results based on DNA sequences are shown in Fig. S4. Results shown are average of three parallel microcosm setups. Standard deviation is based on biological triplicates (triplicate microcosm setups). g = gram wet weight. *nosZ* I = typical *nosZ*, clade I *nosZ*; *nosZ* II = atypical *nosZ*, clade II *nosZ*.

To evaluate the influence of (chemo)denitrification on active microbial nitrogen cycling, we followed gene and transcript copy numbers involved in the different denitrification steps (NO_2_^−^, NO, and N_2_O reduction) during sediment incubation (Fig. 3). This was used to determine the effect of Fe(II)-, nitrate-, and nitrite-amendment on the microbial formation and reduction of N_2_O. Independent of the amendment, the copy numbers for *nirK, qnorB, cnorB*, and atypical *nosZ* (clade II *nosZ*) did not change in the microbially active microcosms during incubation. Only, typical *nosZ* (clade I *nosZ*) was substantially upregulated in the active sediments that were amended with nitrite and Fe(II), whereas it was below the detection limit in all other samples (Fig. 3).

### Consequences of N_2_O formation by (chemo)denitrification on the Fe- and N-cycling microbial community

In order to track the microorganisms that are involved in Fe- and N-cycling under denitrifying and chemodenitrifying conditions in our sediments, we analyzed the general microbial community in Fe(II)- and nitrate-/nitrite-amended microcosms (Fig. S2). We found that the addition of Fe(II), nitrate, and nitrite to the marine sediment and the resulting N_2_O formation caused a general microbial community shift based on DNA (present community) and RNA (active community) analysis (Fig. S2). The overall trends for the changes in the present and active microorganisms during microcosm incubation were similar, i.e. similar taxonomic groups were enriched in the DNA- and RNA-based analysis. Here, we present data for the RNA-based analyses. DNA-based results are provided in the supplementary information (Fig. S4).

The RNA-based 16S rRNA sequence analyses showed that the amendment of Fe(II) and nitrate followed by N_2_O formation through (chemo)denitrification led to the enrichment of active *Defluviicoccus* (*Rhodospirialles*) (1.3%)*, Sulfurimonas* (*Campylobacterales*) (21.1%), and *Arcobacter* (*Campylobacterales*) (13.7%) (Fig. S2). *Defluviicoccus sp*. is a glycogen-accumulating organism and typically active in enhanced biological phosphorus removal-activated sludge systems^32^. While *Sulfurimonas* sp. are known for their ability of catalyzing chemolithotrophic reactions with ferrous iron and pyrite and the reduction of nitrate and nitrite^33^, *Arcobacter* sp. are known for their activity in Fe-rich habitats^34^, their ability to catalyze Fe(III) and Mn(IV) reduction^35^, their use of Fe(III) citrate as electron acceptor^36^, and their nitrogen-fixation ability^37^.

In contrast, Fe(II) and nitrite amendment followed by N_2_O formation through (chemo)denitrification led to the enrichment of active *Psychrilyobacter* (*Fusobacteriales*) (6.3%)*, Propionigenium* (*Fusobacteriales*) (2.2%), *Bacillus* (0.8%), *Thauera* (0.9%), and in particular *Marinobacter* (*Alteromonadales*) (22%) (Fig. S3). *Psychrilyobacter* is known as a psychrophilic Fe(III) reducer and was enriched in ferruginous marine sediments^38^. We identified abundant and active *Marinobacter* (22%) to be closely related to *M. litoralis* (100% 16S rRNA gene sequence similarity), to *M. aquaeolei* (99%; a potential neutrophilic Fe(II)-oxidizer), and to *M. hydrocarbonoclasticus* (99%). Relatives of *M. hydrocarbonoclasticus* were also identified recently based on *nosZ* gene sequence analysis in non-amended Norsminde Fjord sediment (Otte *et al*., unpublished). *M. hydrocarbonoclasticus* has the genetic potential for all nitrogen cycle enzymes and the potential for N_2_O formation^39^ while a close relative, i.e. *M. aquaeolei*, even has the potential for Fe(II) oxidation^40^.

### Evidence for the importance of chemodenitrification on N_2_O formation

To distinguish between N_2_O formation by abiotic chemodenitrification and by microbially catalyzed processes, we inactivated the microbial community in the sediment by gamma-radiation. Gamma sterilization destroys enzymes (RNAses) that are required for RNA degradation^41^. However, even in sterilized samples traces of RNA were found (Fig. 3), which might result from incomplete sterilization. Still, the sterilized samples can be considered as a valid control setup. In order to evaluate the nitrate-reducing Fe(II)-oxidizing and the Fe(III)-reducing potential of the remaining RNA in the sterilize sediments, participation in nitrogen-converting metabolisms was checked using the *KEGG database* (*KEGG pathways*). Based on a previous strategic study on the efficiency of sterilization we can still consider gamma-radiation as the most efficient sterilization method for sediment samples^41^.

## Discussion

Our results show that chemodenitrification can account for up to 15–25% of the total N_2_O production in the marine sediments from Norsminde Fjord (Fig. 1). High N_2_O release in the presence of Fe(II) and nitrate/nitrite has been observed before in different environments^27,30,42^. For Norsminde Fjord previous studies reported N_2_O concentrations at the sediment-water/atmosphere interface of up to 0.49–4.9 µM^43–45^. Although, N_2_O emission rates are in a similarly high range compared to our data, the production of N_2_O has never been related to chemo-denitrification. The source of N_2_O formation has mainly been described to originate from heterotrophic denitrification or mixotrophic nitrate-dependent Fe(II) oxidation^26^. For the latter process, it has recently been shown that the oxidation of Fe(II) in the presence of nitrate and dissolved organic carbon, is a coupled abiotic-biotic reaction network (e.g. in *Acidovorax* sp. BoFeN1^12^). This means that nitrate gets microbially (heterotrophic) reduced and intermediate reactive nitrogen species (e.g. nitrite) are involved in chemodenitrification which produces N_2_O upon the oxidation of Fe(II)^12^. Few studies hypothesized that chemodenitrification might play a much bigger role in the N_2_O production patterns in ecological systems than previously thought^25,30^. Wankel *et al*.^30^ showed that N_2_O increases in stratified marine coastal sediments as soon as oxygen is consumed along the redox gradient. These results in combination with our data imply the importance of N_2_O production due to chemodenitrification.

**Figure 4:**
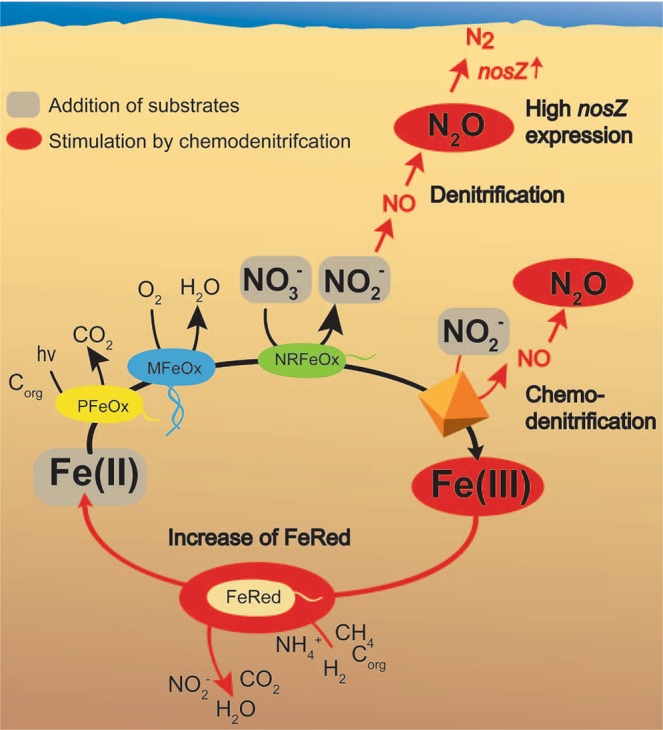
Cause and effects of nitrite- and nitrate-induced (chemo)denitrification-based N_2_O formation in coastal marine sediment. When Fe(II) and nitrite/nitrate was added to the marine sediment, Fe(III) was formed among different process by chemodenitrification which is stimulating Fe(III)-reducing bacteria (FeRed). The FeRed bacteria then produce Fe(II) and stimulate Fe(II)-oxidizing bacteria. Therefore chemodenitrification has a significant impact on Fe-cycling in general. Simultaneously, nitrite/nitrate was reduced to NO and further to N_2_O by chemodenitrification and denitrifying bacteria. In addition, the presence of high nitrite/nitrate concentration leads to a high typical *nosZ* gene expression in denitrifying bacteria which is responsible for the reduction of N_2_O into N_2_.

Anoxic conditions influence the expression of genes involved in the reduction of N oxides (Figs 3 and 4). Although the presence of elevated N_2_O concentrations was thought not to upregulate the expression of *nosZ* genes (within clade I of *nosZ*-harboring bacteria)^46^, recently, Harter *et al*.^47^ suggested that the increase of typical *nosZ* expression under nitrate-reducing conditions might be due to locally enriched N_2_O. In our study, 46% more N_2_O was formed (after 91.5 hours of incubation) in microbially active microcosms that were amended with Fe(II) and nitrite (Fig. 1), compared to the microcosms that were amended with Fe(II) and nitrate only. In these microcosms elevated expression of typical *nosZ* was detected (Fig. 3). High N_2_O concentrations, as well as high nitrite levels, were described to have a toxic effect on the microorganisms, which might trigger the expression of typical *nosZ* genes as a detoxification mechanism^48–51^. The measured N_2_O and nitrite levels in our experiments only represent the net concentrations, and potentially the total N_2_O formed is underestimated. Based on thermodynamic considerations, the reduction of N_2_O to N_2_ provides a higher energy yield than for the other denitrification steps^48,52^. Therefore, microorganisms that are capable of N_2_O reduction (e.g. *Shewanella* spp., *Marinobacter* spp., and *Pseudomonas* spp., which were enriched and active in our microcosms (Fig. S3)), might gain an energetic advantage over species performing the full denitrification pathway (with *nirK/S*, *nosZ*)^53,54^. This advantage is only available as long as anoxic and no sulfidic conditions are prevailing, as the presence of sulfide and oxygen can inhibit the expression of *nosZ* genes and the concurrent reduction of N_2_O to N_2_. Recently, a second clade of *nosZ* (atypical *nosZ*; clade II *nosZ*) was discovered and atypical *nosZ* were shown to be expressed in non-denitrifying N_2_O-reducing microorganisms^49,54^. Hallin *et al*.^49^ proposed that clade II bacteria have a N_2_O respiratory chain that allows more efficient free energy conservation compared to the clade I system^49^. The energetic benefit from N_2_O reduction might explain the high expression of atypical *nosZ* in all setups. However, only bacteria with the full denitrification capacity are upregulating typical *nosZ* in the setup where high N_2_O concentrations (due to chemodenitrification) accumulate to cytotoxic levels^47^.

Our data show that nitrite-induced chemodenitrification has substantial consequences for the active N- and Fe-cycling microbial community (based on 16S rRNA gene sequences) (Figs 4 and S2, S3). Community members that carry the potential to trigger heterotrophic Fe(III) and nitrate reduction were enriched and active (Fig. S3), which is also supported by the slight decrease of DOC in nitrate and Fe(II) amended microcosms (Fig. 1). The stimulating effects of chemodenitrification on active Fe-cycling microorganisms (based on 16S rRNA gene sequences) were obvious by an increase of potential Fe(III)-reducers (*Arcobacter, Sulfospirillum*, and *Shewanella* in nitrate-amended setups, and *Psychrilyobacter* in nitrite-amended setups). In addition, an increase in the relative abundance of active Fe(II)-oxidizing bacteria [*Sulfurimonas* (with nitrate amendment), and *Marinobacter* and *Pseudomonas* (with nitrite amendment)] has been confirmed by 16S rRNA gene sequence analysis (Fig. S3)). Several *Marinobacter* species have been found in Fe-rich habitats^55^, and their metabolic potential to utilize nitrate as terminal electron acceptors and iron (i.e. FeS_2_ and CuFeS_2_) as an electron donor has been demonstrated previously^40,56^. Potential Fe(III)-reducers (e.g. *Desulfobulbus*, *Desulfomusa, Sulfospirillum, Shewanella*) were more abundant and active than bacteria that might be enrolled in Fe(II) oxidation. These results are in line with previous studies that quantified significantly more Fe(III)-reducing bacteria (up to 2.8%) compared to Fe(II)-oxidizing bacteria (in particular nitrate-reducing Fe(II)-oxidizers with 0.3%) in the same sediment^57,58^. Potential denitrifying bacteria with typical *nosZ* such as *Shewanella* spp. (which are metabolically flexible, i.e. they can use either Fe(III), nitrate or nitrite, and were detected in nitrate-amended microcosms), *Pseudomonas* spp. and *Marinobacter* spp. (in nitrite-amended microcosms) were stimulated by nitrate/nitrite addition and chemodenitrification reactions (Fig. S3, selection of typical *nosZ* bacteria from Norsminde Fjord sediment).

The surviving community (*Xanthomondales* clade JTB255, Candidatus *Isobeggiatoa divolgata*, *Oceanospirillales*, *Sandaracinaceae*, and *Bacteroidetes* at t_0_) and enriched microorganisms (e.g. *Synechococcus* and *Cyanobium* sp. at t_end_) in the gamma-sterilized microcosms (analyzed at both t_0_ and t_end_) (Fig. S2) have the ability of assimilatory nitrate reduction (reduction of nitrate to nitrite) but no genetic potential (e.g. no *nirK/S* and no *norB* genes) for further nitrite reduction. In addition, we did not see an increase of nitrite (Fig. 1) in microbially inactivated sediment. We can therefore conclude that all N_2_O produced in gamma-radiated sediments can be attributed to an abiotic mechanism, namely chemodenitrification.

In this study we observed that up to 15–25% of total N_2_O production might be caused by chemodenitrification, whereas 75–85% of N_2_O was produced by denitrification and other microbial processes. 1.2 mM dissolved Fe(II) was consumed in microcosms with sterilized sediment, that were amended with 2 mM Fe(II) and 4 mM NO_2_^−^ (Fig. 1). Following the stoichiometry of Equation 1, 1.2 mM Fe(II) can abiotically reduce 0.6 mM NO_2_^−^. The measured decrease in NO_2_^−^ was approx. 0.5 mM NO_2_^−^ (Fig. 1), and we can therefore consider chemodenitrification as the main process in the microcosm setup with sterilized marine sediment. In the microbially active setups 1.4 mM Fe(II) and 1.3 mM NO_2_^−^ was consumed. Based on the stoichiometry of chemodenitrification the consumption of only 0.7 mM NO_2_^−^ can be attributed to this process. The consumption of the remaining 0.7 mM NO_2_^−^ can be related to various microbial processes in the sediment.

However, transferring our data to environmental systems we need to keep in mind several rate limiting factors for chemodenitrification. Compared to *in situ* conditions our setups do not suffer from substrate limitations, i.e. Fe^2+^ and nitrite production rates. Also, Fe-organic matter complexes, as they potentially occur in nature, will influence the reaction kinetics and the fate of Fe and N species^59^. In addition to that, in nature other processes, such as anammox, DNRA, and nitrate reduction by sulfur oxidation will impact the N_2_O emission rate^60,61^. Finally, the incubation temperature of 25 °C represents conditions that can be reached in summer months, especially when the overlying water column is very shallow^62,63^. Although these conditions do not represent average field site conditions over the whole year, they represent the environmental circumstances at which maximum N_2_O emission via chemodenitrification can be expected.

Coastal marine environments have been recognized as an important source of N_2_O to the atmosphere^28,29^. Sandy sediments, such as the one we used for our study, are representative for about 70% of all global shelf sediments^64^ and are expected to release up to 1.9 Tg N_2_O-N yr^−1^ ^28^. Assuming a contribution of 15–25% N_2_O release by chemodentrification (data from this study) shelf sediments might account for 0.3–0.5 Tg N_2_O-N yr^−1^ abiotically produced N_2_O. In the future the N_2_O release might even increase due to higher fertilizing activities in close-by agricultural fields. Leaching and runoff transport of nitrate into oceans leads to coastal marine eutrophication^65^ and increases the potential for additional N_2_O production by (chemo)denitrification. There is a high chance that similarly high N_2_O emission rates that have been observed in many other environments, including soils^26^ and sediments^28–30^, are triggered by chemodenitrification.

## Methods

### Site description and sampling procedure

Marine sediment samples were taken in February 2015 April 2016, and March 2017 from the Aarhus Bay area (Denmark) (geochemical characterization is described in Laufer *et al*.^58^ and Table 1). Sediment from the shallow marine estuary Norsminde Fjord (NS) was collected at 0.5 m water depth near its narrow entrance from Aarhus Bay (N 56°01.171′; E 010°15.390′). Bulk sediment from the upper 3 cm were collected, transported to the laboratory at 4 °C, and the sediment was stored for a few weeks at 4 °C until microcosm experiments were set up.

**Table 1:** Geochemical parameters of sediment from Norsminde Fjord in Denmark.

Geochemical parameters	
Salinity	14.6‰
pH anoxic porewater	7.2
O_2_ penetration depth	3.2 mm
Light penetration depth	2.2 mm
Fe(II)_diss_ in sediment	73 ± 28 µmol g^−1^ dw*
Fe(II)_diss_ in porewater	29 ± 4.9 µM*
Fe(II)_total_ in porewater	106 ± 11 µM*
Nitrate (in porewater)	18.3 ± 8.2 µM*
Nitrite (in porewater)	*Below detection limit*
Sulfide (in porewater)	*Below detection limit*
DOC (in porewater)	3.9 ± 0.1 mg l^−1^*
TIC (in porewater)	28.9 ± 0.1 mg l^−1^*

Detailed sediment geochemistry description see Laufer *et al*.^8^. *Mean value for measurements in the upper 3 cm of the sediments.

### Geochemical analyses

Temperature, pH, salinity, and oxygen concentration of the water column were analyzed in the field with a multimeter (WTW, Multi 3430). The geochemical parameters of sediment water content, DOC, and nitrate concentrations in porewater as well as Fe(II) and Fe(III) concentrations in porewater and sediment were determined in the laboratory. All analyses are described in detail in Laufer *et al*.^58^.

### Microcosm experiment

Bulk sediment from Norsminde Fjord was homogenized before setting up microcosm incubations. Microcosm incubations were set up in 100 ml serum vials that were wrapped in aluminum foil for dark incubations. Five g of homogenized bulk sediment and 50 ml anoxic filtered seawater were used for each microcosm. The headspace of the microcosms was N_2_/CO_2_ (90:10). For preparation of the media, seawater was made anoxic by flushing with N_2_ for at least 1 h per liter, filtering it through a 0.22 µm filter (EMD Millipore Steritop^TM^) and replacing the headspace by N_2_/CO_2_ (90:10), followed by adding 20 mM NaHCO_3_ as buffer (final pH of the microcosms: 7.2 ± 0.1). To inhibit the activity of sulfate-reducing bacteria (and therefore inhibit reactions of sulfur species with Fe(II)) an anoxic and sterile filtered 1 M Na_2_MoO_4_ solution was added to a final concentration of 20 mM^58^. The pH of the water was adjusted to 7.1 and regularly checked during incubation. The microcosms were amended with different substrates: (i) 2 mM Fe(II) (FeCl_2_) and 4 mM NO_3_^−^ (NaNO_3_^−^), (ii) 2 mM Fe(II) (FeCl_2_) and 4 mM NO_2_^−^ (NaNO_2_^−^), and (iii) 4 mM NO_2_^−^ only, each set of substrates added to native and sterilized sediment. The different setups are summarized in Table S1.

Abiotic control microcosms with gamma-sterilized sediment were incubated under the same conditions as the microbially active ones. Sediment for gamma-sterilization (sterile microcosm experiments) was filled into plastic bags, sent to Synergy Health Allershausen, Germany, and radiated at 52 kGy with 5% radiation tolerance. All microcosm experiments were setup in triplicates and incubated in dark at 25 °C. The chosen temperature represents conditions at which the observed processes will have a maximum environmental impact. Although the yearly average temperature in the field is significantly lower, the chosen temperature will be reached in summer months, especially when the water column is very shallow^62,63^.

### Quantification of Fe(II), nitrate, nitrite, and DOC

For measuring Fe(II) and extractable Fe, 1 ml of slurry from each microcosm was sampled with a syringe inside an anoxic glovebox (100% N_2_ atmosphere). 100 µl of this slurry sample was added to 900 µl 40 mM sulfamic acid in 1 M HCl and placed on a shaker (150 rpm) for 1 h (see Laufer *et al*.^58^). The samples were then centrifuged (5 min, 7000 g, Eppendorf 5430R) and the supernatant was used for Fe(II) and extractable Fe determination with the spectrophotometric Ferrozine assay^21^. The remaining part of the initial 1 ml sample of the slurry was centrifuged and the supernatant was used for analyses of the dissolved phase. The Fe-extraction with sulfamic acid^21^ avoids the abiotic oxidation of Fe(II) by nitrite during acidic Fe-extraction. For nitrate and nitrite, 100 µl of supernatant was added to 900 µl Milli-Q H_2_O and stored anoxically at 4 °C until analysis by flow injection analysis (FIA)^58^. For analysis of DOC in microcosm incubations, 20 ml of sample was necessary^58^ and the contents of sacrificial microcosms were centrifuged for 15 min at 5000 g (Hermle 7300 Germany). Afterwards, the supernatant was filtered through a 0.45 µm filter (MF-Millipore MCE membrane) and the DOC concentration was measured with a carbon analyzer (High TOC, Elementar, Germany).

### Nitrous oxide quantification

For the determination of N_2_O concentrations, the pressure of the microcosm bottles was brought to normal pressure (1 bar) before sampling via a water trap. Headspace gas samples of 0.5 ml were taken from the microcosms 0, 15, 39, 63, and 87 hours after the beginning of the incubation and transferred into 22.5 ml evacuated gas chromatograph (GC) vials using a gas-tight syringe (1100TLL 100 ml Gastight, Hamilton, Reno, NV, USA). The trace gas concentrations in the GC vials (22.5 ml) were measured using a GC equipped with an electron capture detector (^63^Ni-ECD) for N_2_O and CO_2_ (Hewlett Packard, 5890 Series II). The GC setup and configuration was described in detail previously^66^. Gas fluxes were calculated using the slope of the temporal change in concentration of the closed microcosms according to the equations published in Ruser *et al*.^66^. The result of 17% of total N_2_O by chemodenitrification is an average of three independent experiments and we show results of only one experiment.

### DNA and RNA extraction, DNA digestion, reverse transcription, and amplification

Total DNA and RNA was extracted using the PowerSoil® RNA and DNA isolation kit as directed by the manufacturer (MO BIO Laboratories, Carlsbad, CA, USA), with the following modifications: 0.8 g to 2 g sediment was used from each sediment slice; 5 min bead-beating; centrifugation steps at maximal speed (7000 g) at 4 °C; and a longer incubation times at −20 °C (1.5–2.0 hours). RNA and DNA were eluted in 50 µl 10 mM Tris buffer. DNA and RNA concentrations were determined using a Qubit® 2.0 Fluorometer with DNA and RNA HS kits (Life Technologies, Carlsbad, CA, USA). RNA extracts were digested with the Ambion Turbo DNA-free^TM^ kit as directed by the manufacturer (Life technologies, Carlsbad, CA, USA). Successful DNA removal was confirmed by PCR using general bacterial primers (see Supplementary Information). Subsequent reverse transcription reactions were done using a reverse transcriptase (Invitrogen, Life Technologies) as described in the supporting information. Microbial 16S rRNA genes were amplified using primers 515F and 806R (see Supplementary Information) targeting the V4 region. Quality and quantity of the purified amplicons were determined using agarose gel electrophoresis and Nanodrop (NanoDrop 1000, Thermo Scientific, Waltham, MA, USA). Subsequent library preparation steps and sequencing were performed by Microsynth AG (Balgach, Switzerland). A sequence library was prepared and sequence adapters added using the Nextera kit. Sequencing was performed on an Illumina MiSeq sequencing system (Illumina, San Diego, CA, USA) using the 2 × 250 bp MiSeq Reagent Kit v2 (500 cycles kit) (Illumina, San Diego, CA, USA). The MiSeq reporter software v2.6 (Illumina, San Diego, CA, USA) was used for signal processing, de-multiplexing, and trimming of adapter sequences. The quality of the reads was checked with the software FastQC version 0.11.5 and the primers trimmed using cutadapt v1.14. Amplicon reads (accession number: SRP132652) have been deposited in the NCBI Genbank database (bioproject: PRJNA431287).

### Sequence analysis

Demultiplexed and trimmed reads were further analyzed using QIIME (v1.9.1) (Caporaso reference). Paired end reads were joined using default settings and were further quality filtered and only those with a minimum Phred quality score of Q20 were used. Chimeric sequences were identified using usearch6.1^67^ and removed. OTUs were picked using the QIIME workflow script “pick_de_novo_otus.py” and taxonomically identified at 97% similarity using the SILVA 128 reference database^68^. Singletons were removed from the OTU table prior to further analysis. We used the quality-filtered reads for the graphs (Figs S2 and S3) and checked also with <0.1% filtered OTU tables (data not shown). Rarefaction curves, diversity indices (Shannon diversity, Simpson diversity), richness (Chao1, ACE), and coverage estimators (Good’s coverage) were calculated using QIIME workflow scripts.

### Quantitative PCR of bacterial 16S rRNA genes and nitrogen cycle genes

Quantification PCR (qPCR) specific for phylogenetic and functional marker genes [16S rRNA gene (Bacteria), *amoA* (Archaea), *nirK* (nitrite reductase), *qnorB* and *cnorB* (NO reductases), typical *nosZ* (clade I N_2_O reductase in denitrifiers) and atypical *nosZ* (clade II N_2_O reductase in non-denitrifiers)] was carried out using the SsoFast EvaGreen Supermix (Bio-Rad Laboratories, Hercules, CA, USA), an iQ5 real-time PCR detection system (iQ5 optical system software, version 2.0, Bio-Rad Laboratories), and gene-specific primers. For details on plasmid standards, gene-specific qPCR primers, reaction mixtures and thermal programs, please refer to Table S2 in the Supplementary Information.

## Supplementary information

Supporting information

## Data Availability

The microcosm datasets generated during and/or analyzed during the current study are available from the corresponding author on reasonable request.
