# Supplementary material for: N2O formation by nitrite-induced (chemo)denitrification in coastal marine sediment
Source: Sci Rep. 2019 Jul 31;9:10691. doi: 10.1038/s41598-019-47172-x (PMC6668465; doi:10.1038/s41598-019-47172-x)
Supplement: Supplementary file 1 — Supporting information [file 41598_2019_47172_MOESM1_ESM.docx]

**N_2_O formation by nitrite-induced (chemo)denitrification in coastal marine sediment**

*Julia M. Otte^1^, Nia Blackwell^1,2^, Reiner Ruser^3^, Andreas Kappler^1,4*^, Sara Kleindienst^1,2^,
Caroline Schmidt^1^*

^1^ Geomicrobiology, Center for Applied Geosciences, University of Tübingen, Germany

^2^ Microbial Ecology, Center for Applied Geosciences, University of Tübingen, Germany

^3^ Fertilization and Soil Matter Dynamics, Institute of Crop Science, University of Hohenheim, Germany

^4^ Geomicrobiology, Center for Geomicrobiology, Aarhus University, Denmark

**Supplementary information**

**Supplementary methods**

**Plasmid standards, gene-specific qPCR primers, reaction mixtures and thermal programs**. RNA extracts were digested with the Ambion Turbo DNA-freeTM kit as directed by the manufacturer (Life technologies, Carlsbad, CA, USA). Successful DNA removal was confirmed by PCR using general bacterial primer GM3-8f and 1392R^1,2^. Microbial 16S rRNA genes were amplified using primers 515F (5′-AATGATACGGCGACCACCGAGATCTACACTATGGTAATTGTGTGCCAGCMGCC-GCGGTAA-3′) and 806R (5′-CAAGCAGAAGACGGCATACGAGATAGTCAGCCAGCCGGACTACNVGGGT-WTCTAAT-3′)^3^ targeting the V4 region^3^. PCR mixtures used for amplification contained per reaction: 0.5 μl of 10 μM of each primer (515F and 806R), 12.5 μl of 2x KAPA HiFi HotStart Readymix (Kapa Biosystems, Inc., Wilmington, MA, USA) and 9 μl of RNAse/DNAse-free water and 2.5 μl template (cDNA or DNA). The following thermal profile was used: 3 min at 95 °C, 30 cycles of 95 °C 30 s, 55 °C 30 s, 72 °C 30 s and 5 min at 72 °C. Quality of the amplified DNA and cDNA was confirmed on an Experion® automated electrophoresis system using an Experion® DNA analysis kit (Bio-Rad Laboratories, Hercules, CA, USA).

Bacterial strains and primers which were used for the construction of qPCR-standards (16S rRNA, *nirK*, typical *nosZ*) can be found in the Supplementary material of Harter *et al*., 2014 (Table S2)^4^ and atypical *nosZ* clade II in Harter *et al*., 2016 (Table S1)^5^, as well as quantitative PCR reaction mixtures and thermal profiles for the different target genes of 16S rRNA, *nirK* and *nosZ* (Table S3^4^ and Table S2^5^). For *qnorB* qPCR standards (origin: *Ralstonia eutropha*) and primers pairs 2F/5R and cnorB qPCR standards (origin: *Pseudomonas aeruginosa*) and primers pairs 2F/6R were used (Table S1). The qPCR thermal program for bacterial primers 341F (5`-CCTACGGGAGGCAGCAG-3`)^1^ and 797 R (5`-GGACTACCAGGGTATCTAA-TCCTGTT-3`)^6^ was: 2 min at 98 °C, 40 cycles of 5 s at 98 °C and 12 s at 60 °C and for *cnorB* and *qnorB* see Table S1. Each sample was quantified in duplicates using the iCycler iQ Real-Time PCR Detection System and the iQ 5 Optical System software, version 2.0 (Bio-Rad laboratories). During qPCR setup, evaluation and data analysis, we followed the MIQE guidelines^7^.

**Supplementary table**

**Table S1: Overview of all performed microcosm experiments.** Triplicate microcosm setups of natural and sterile marine sediments (from three different sampling campaigns: 2015-2017) with no additives, only NO_2_^-^, NO_3_^-^ + Fe(II), or NO_2_^-^ + Fe(II).

| **Date of experiment (month and year)** | **Sampling campaign** | **Natural or sterilized sediment** | **Substrate addition**  **(+ different dilutions)** |
| --- | --- | --- | --- |
| April 2016 | March 2016 | Natural | NO_3_^-^ + Fe(II) |
|  |  | Sterile | NO_3_^-^ + Fe(II) |
|  |  | Natural | NO_2_^-^ + Fe(II) |
|  |  | Sterile | NO_2_^-^ + Fe(II) |
| April 2017 | March 2017 | Natural | - |
|  |  | Sterile | - |
|  |  | Natural | NO_2_^-^ |
|  |  | Sterile | NO_2_^-^ |

**Table S2: *qnorB* and *cnorB* primers used for qPCR.**

| **Target gene** | **Primer name** | **Primer sequence**  **(5`-3`)** | **Fragment size** | **Reference** | **Origin of standard/**  **strain** | **Thermal profile** |
| --- | --- | --- | --- | --- | --- | --- |
| *cnorB* | *cnorB* 2F  *cnorB* 6R | GAC AAG NNN TAC TGG TGG T  GAA NCC CCA NAC NCC NGC | 393 bp | Casciotti and Ward, 2005;  Jung *et al*., 2002 | *Pseudomonas aeruginosa* | 98 °C – 5 min  95°C – 30 s  57°C – 40s  72°C – 1 min  X 40 cycles |
| *qnorB* | *qnorB* 2F  *qnorB* 5R | GGN CAY CAR GGN TAY GA  ACC CAN AGR TGN CAN ACC CAC CA | 266 bp | Baker *et al*., 2003 | *Ralstonia eutropha* | 98 °C – 5 min  95°C – 30 s  57°C – 40s  72°C – 1 min  X 40 cycles |

**Supplementary figures**

Natural sediment

Sterile sediment

Natural sediment + Nitrite

Sterile sediment + Nitrite


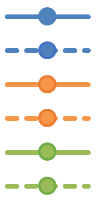


**Fig. S1 Sediment microcosms without amendment and with amendment of nitrite.** Microcosms contain either natural sediment only or were amended with 4 mM nitrite (microbial active vs. sterilized marine Norsminde Fjord sediment) collected in spring 2017. Results shown are average of three parallel microcosm setups.

**
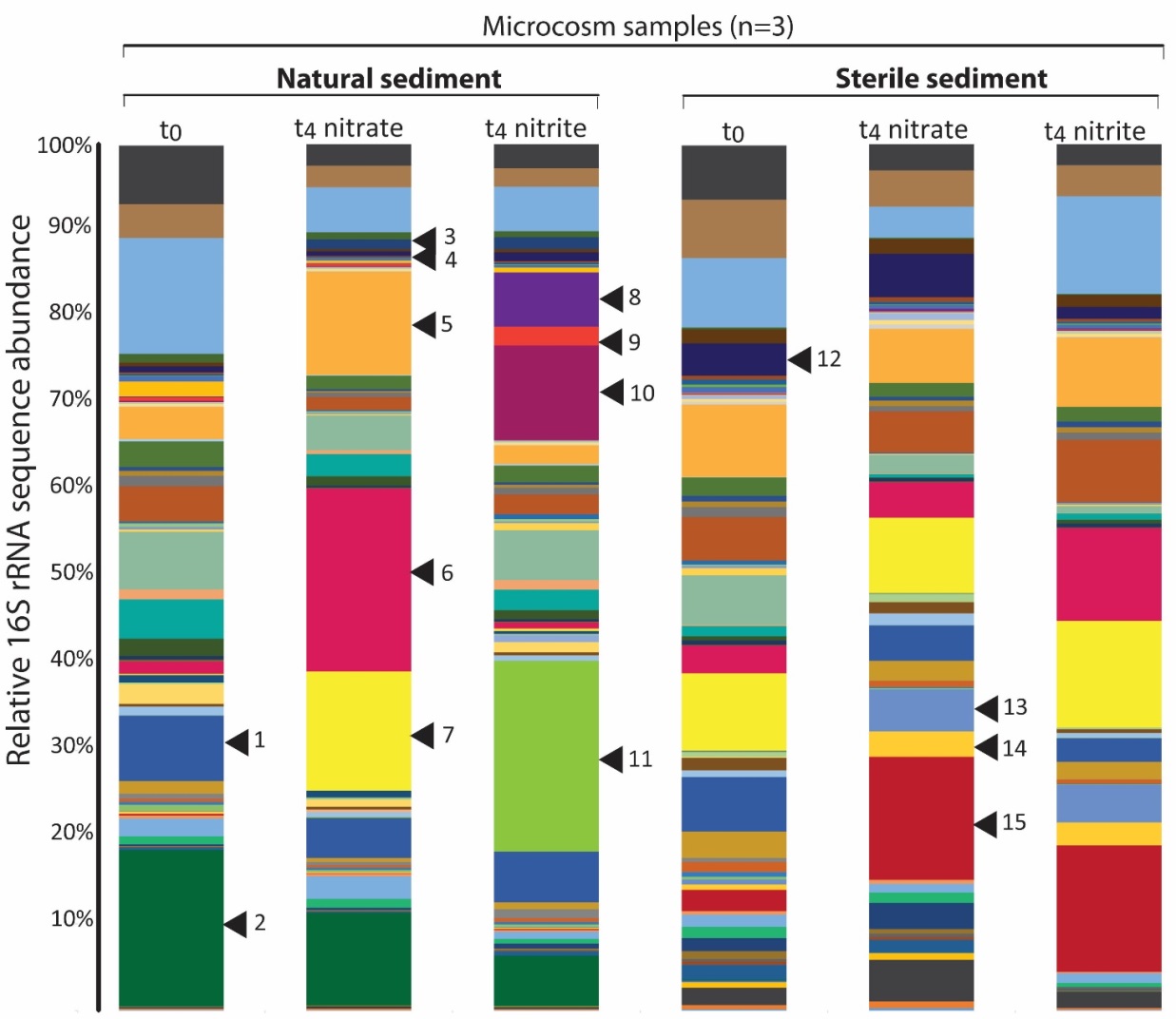
**

**Fig. S2 Microbial community composition at t_0_ and t_4_ (after 87 hours) based on 16S rRNA sequences.** Relative 16S rRNA sequence abundance based on RNA from natural sediment and sterile sediment (abiotic control). 1) *Proteobacteria*, *Thiobios*, 2) *Cyanobacteria*, uncultured diatom, 3) *Rhodospirialles, Defluviicoccus*, 4) Uncultured *Rhodocyclales*, 5) *Desulfuromonadales*, 6) *Campylobacterales, Sulfurimonas*, 7) *Campylobacterales, Arcobacter*, 8) *Fusobacteriales, Psychrilyobacter,* 9) *Fusobacteriales, Propionigenium*, 10) Uncultured *Fusobacteriales*, 11) *Alteromonadales, Alteromonadaceae* which includes *Marinobacter* spp., 12) *Myxococcales, Sandaracinaceae*, 13) Uncultured *Synechococcales*, 14) *Synechococcales, Cyanobium* sp. SDEC6, 15) *Synechococcales* subsection I.


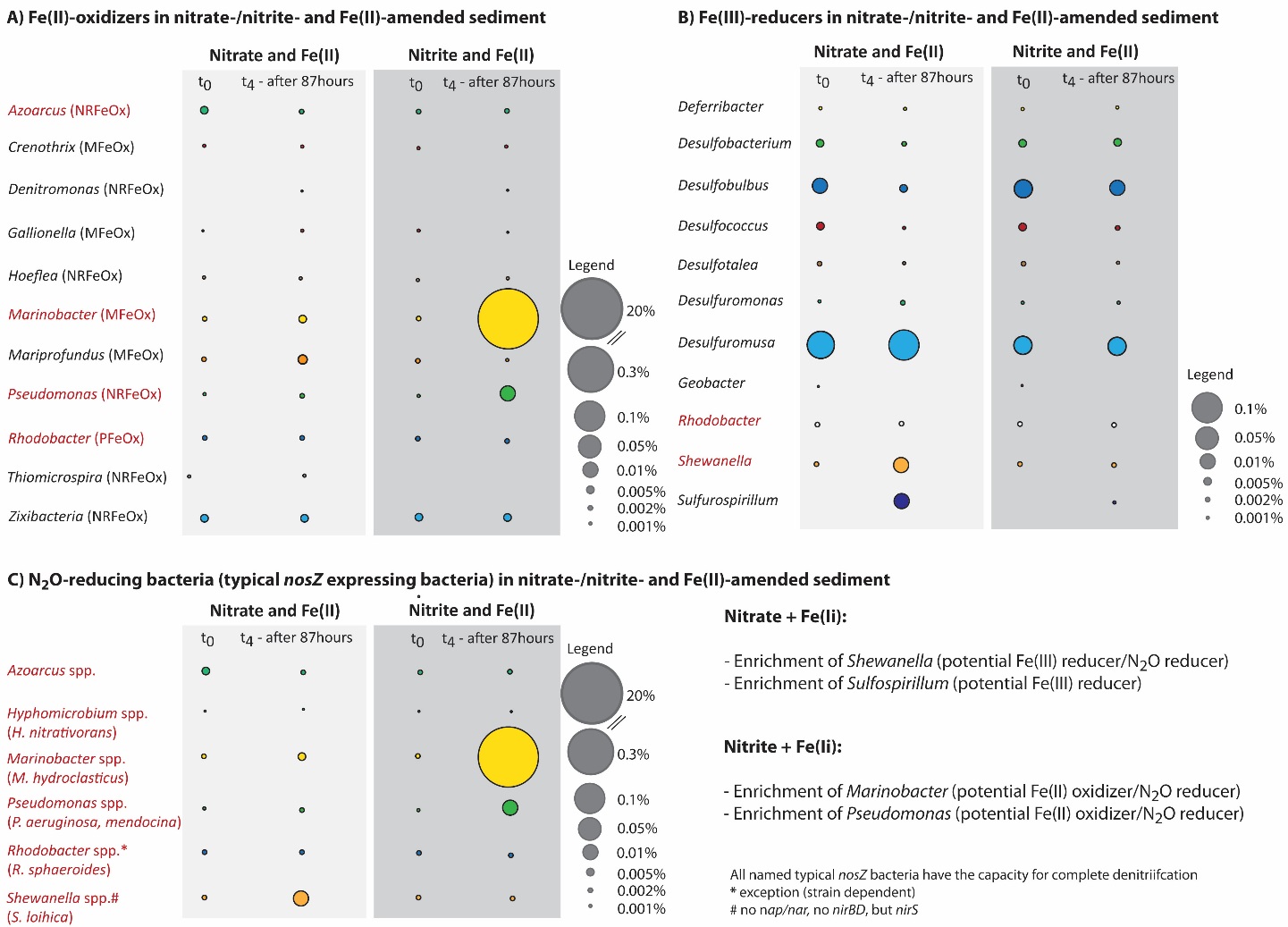


**Fig. S3 Selection of potential Fe(II)-oxidizing (FeOx), Fe(III)-reducing (FeRed) microorganisms and N_2_O-reducing bacteria** at t_0_ and at t_4_ (after 87 hours). Selection of *nosZ* bacteria are from typical *nosZ* gene amplicon sequencing of natural untreated Norsminde Fjord sediment (see Otte *et al*., 2018 ^8^).


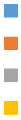


Natural sediment + Nitrate + Fe(II)

Sterile sediment + Nitrate + Fe(II)

Natural sediment + Nitrite + Fe(II)

Sterile sediment + Nitrite + Fe(II)

**Fig. S4 Absolute abundance of bacterial 16S rRNA and nitrogen cycle genes (based on DNA) in biotic and abiotic microcosms (shown in Fig. 1).** Genes in abiotic experiments were detectable. Results based on RNA sequences are shown in Fig. 4. Standard deviation is based on biological triplicates (triplicate microcosm setups). g = gram wet weight. nosZ I = typical nosZ, clade I nosZ; nosZ II = atypical nosZ, clade II nosZ.

**References**

1. Muyzer, G., Teske, A., Wirsen, C. O. & Jannasch, H. W. Phylogenetic relationships of *Thiomicrospira* species and their identification in deep-sea hydrothermal vent samples by denaturing gradient gel electrophoresis of 16S rDNA fragments. *Arch. Microbiol.* **164**, 165-172 (1995).

2. Stahl, D. A., Flesher, B., Mansfield, H. R. & Montgomery L. Use of phylogenetically based hybridization probes for studies of ruminal microbial ecology. *Appl. Environ. Microb.* **54**, 1079-1084 (1988).

3. Caporaso, J. G.*, et al.* QIIME allows analysis of high-throughput community sequencing data. *Nat. Methods* **7**, 335-336 (2010).

4. Harter, J.*, et al.* Linking N_2_O emissions from biochar-amended soil to the structure and function of the N-cycling microbial community. *ISME J.´* **8**, 660-674 (2014).

5. Harter, J.*, et al.* Gas entrapment and microbial N_2_O reduction reduce N_2_O emissions from a biochar-amended sandy clay loam soil. *Sci. Rep.* **6**, 39574 (2016).

6. Nadkarni, M. A., Martin, F. E., Jacques, N. A. & Hunter, N. Determination of bacterial load by real-time PCR using a broad-range (universal) probe and primers set. *Microbiol.* **148**, 257-266 (2002).

7. Bustin, S. A.*, et al.* The MIQE guidelines: minimum information for publication of quantitative real-time PCR experiments. *Clinic. Chem.* **55**, 611-622 (2009).

8. Otte, J., Harter, J., Laufer, K., Blackwell, N., Kappler, A., Kleindienst, S. The distribution of active iron-cycling bacteria in marine and freshwater sediments is decoupled from geochemical gradients. *Envion. Microbiol.* **20**, 2483-2499 (2018).
